# A new species of *Protonemura* Kempny, 1898 (Plecoptera, Nemouridae) from Albania

**DOI:** 10.3897/BDJ.12.e129725

**Published:** 2024-08-21

**Authors:** Pia Teufl, Wolfram Graf

**Affiliations:** 1 Institut für Hydrobiologie und Gewässermanagement, Wien, Austria Institut für Hydrobiologie und Gewässermanagement Wien Austria

**Keywords:** Albania, Bënçë, new species, *Protonemuracorsicana* species group, *Protonemuraeclipsis*, Tepelenë District

## Abstract

**Background:**

Although studies of the entomofauna of the Balkan Peninsula have increased in quantity and intensity over the course of the last decades, many areas are still not fully investigated regarding their faunistic inventory.

**New information:**

As a result of a field trip in the Vjosa catchment in 2023, a new species of the genus *Protonemura* Kempny, 1898 (Plecoptera, Nemouridae, Amphinemurinae) is described from Albania, *Protonemuraeclipsis* sp. nov. from a holotype male, collected from the District of Tepelenë, Bënçë River. The new species is compared and differentiated from related species, namely *Protonemuraalbanica* Raušer, 1963 and *Protonemuramiatchense* Ikonomov, 1983. It differs from congeners clearly by its terminalia, namely the shape of the paraprocts. Images of the new species and of *Protonemuraalbanica* are provided.

## Introduction

The species inventory of a specific area is shaped by a multitude of factors acting on different temporal and spatial scales. On a geological time scale, prehistoric events like orogenesis or glaciation play a major role, while on site-scale, physico-chemical parameters, hydromorphological conditions, but also food availability and competition act as main parameters for species distribution and diversity (Graf et al. 2018). The Balkan Peninsula is largely known as a biodiversity hotspot (Kryštufek and Reed 2004, Graf et al. 2018, Psonis et al. 2018), including a particularly high number of endemic species (Murányi 2007, Previšić et al. 2014, Graf et al. 2023). The high diversity of the Balkans can be traced back to the complex geological and climatological history of the area (Psonis et al. 2018), which acted as an important refugial territory during the Last Glacial Period (Kühne et al. 2017).

In Albania - part of the Hellenic western Balkan (Illies 1978) - several hydrogeographical and geomorphological specifications additionally contribute to a high habitat heterogeneity and, consequently, a rich faunal inventory. Located between the Dinaric Mountains in the north, the Pindos Mountains in the south and the Adriatic Sea, Albania is rich in freshwaters from springs to potamal habitats which are hydromorphologically still undisturbed and, therefore, in an excellent ecological condition (Murányi 2007). Although faunistic research on the Balkan Peninsula has intensified in recent decades, many areas are still poorly investigated, which also accounts for the order Plecoptera (Murányi 2007, Popijač and Sivec 2009, Petrović et al. 2014, Bilalli et al. 2023). Associated with cold, well-oxygenated waters in mountainous and semi-mountainous rivers (Tyufekchieva and Rimcheska 2019), this group is generally known for its intolerance to environmental degradation (Petrović et al. 2014). As a consequence, Plecoptera are not only a valuable indicator group in environmental monitoring programmes, but also one of the most endangered insect groups in Europe (Fochetti and Tierno de Figueroa 2006).

In 2023, a collecting trip was conducted in the Vjosa catchment, which supports a particularly diverse invertebrate fauna, due to the mostly pristine status of the main river, as well as its tributaries (Graf et al. 2018, Schiemer et al. 2020, Brasseur et al. 2023, Malicky and Graf 2024). The study resulted in the identification of a new species in the genus *Protonemura* Kempny, 1898. This genus belongs to the family Nemouridae, respectively the subfamily Amphinemurinae (Baumann 1975). The presence of an expansion with a bifid opening at the apex of the epiproct (referred to as terminal filament) places the new species in the *Protonemuracorsicana* group, which currently comprises 31 species and three subspecies, divided into four subgroups: *corsicana*, *talboti*, *consiglioi* and *spinulata* (Vinçon and Murányi 2009). The separation of the subgroups is based on characteristics of the male genitalia, such as the outer lobe’s apex, the size and orientation of the terminal filament of the epiproct and the ventral bulge of the epiproct (ibid.). Members of the group are distributed from the Pyrenees to the Caspian Sea; however, only *P.albanica* Raušer, 1963 and *P.miatchense* Ikonomov, 1983 are currently known from the Balkans (Vinçon and Murányi 2009). While *P.albanica* is regarded as endemic to southwest Albania (Murányi 2007), *P.miatchense* is known from western Macedonia only (Ikonomov 1983). Within the *corsicana* group, the new species shows morphological similarities to *Protonemuraalbanica* and is, therefore, compared and distinguished from this species. Morphological differences from *P.miatchense* are mentioned as well.

## Materials and methods

The specimen was collected by an aerial net and stored in 75% ethanol. The holotype is deposited at the Institute of Hydrobiology and Aquatic Ecosystem Management (IHG), Department of Water, Atmosphere and Environment (WAU), University of Natural Resources and Life Sciences, Vienna (BOKU). Photos were made with the Keyence VHX-7000 digital microscope.

## Taxon treatments

### Protonemura eclipsis

Teufl & Graf 2024 sp. nov.

B099D9AD-6F3F-5F34-A5C7-AA34E8A1FC98

C73CBC81-8FFD-4B78-9296-E764ED7E45D9

#### Materials

- **Type status:** Holotype. **Occurrence:** sex: 1 male; lifeStage: adult; occurrenceID: B29114C6-10F6-5D29-A489-C78E94778D96; **Location:** continent: Europe; waterBody: Bënçë river; country: Albania; county: Tepelenë District; verbatimElevation: 303 m.a.s.l; verbatimCoordinates: 40°15'4" (N) 19°57'25" (E); **Event:** samplingProtocol: aerial net; year: 2023; month: 03; day: 26; habitat: mountain river

#### Description

Forewing length 11 mm. Wings yellowish with vigorous dark venation.

Head dark brown; lateral occipital spots light brown; antennae dark brown and approximately as long as the body; palpi pale with dark margins.

Cervical gills long and slender, without apical constrictions.

Pronotum slightly lighter than head; with dark rounded markings; anterior margin dark; lateral margins light brown; quadrangular-shaped with sharp rectangular corners; anterior margin slightly broader than posterior one.

Legs generally yellowish; colouration similar at fore-, mid- and hindlegs; femora yellowish with narrow dark bands at distal ends; tibiae covered with short dark bristles; tarsi dark brown.

Abdominal segments 1-7 middle brown; segments 8-10 dark brown; pronounced pilosity on segments 8-10.

Tergum VIII dark brown, with several dark short posteriomedial spines. Tergum IX sclerotised, with a posteriomedial membranous indentation in between two lobe-like sclerotised structures, bearing several short, black spines. Tergum X sclerotised, except for a wide medial membranous depression bearing several anterolateral spines (Fig. 1b).

Male terminalia (Fig. 1): Hypoproct basally subrectangular; about half the width of sternite IX; longer than wide; with parallel lateral margins; distally extended into a short, blunt process.

Vesicle claviform; more than two times longer than wide.

Paraprocts trilobed. Inner lobe much shorter than median and outer lobes; distally expanding before ending in an elongated, slender apex. Median lobe with wide, rounded base; basal sclerotised structure triangular-shaped with a distinctly pointed apex; prolonged in a pronounced and darkly sclerotised, inwards-orientated inner expansion with several spines at the distal end and a membranous apex covered with setae, approximately as long as the ridge-like inner expansion. Outer lobe in ventral view boot-shaped; widely enlarged at the tip; bearing a strong, inwards-curved spine.

Cerci conical; short; covered with setae; tip blunt, with a small nipple-like structure (Fig. 1a).

Epiproct with parallel margins, narrowing towards the apex. Terminating in a blunt, rounded tip, which is prolonged by a bilobed, membranous extension. Terminal filament rather short and bifid. In lateral view, the filament extends in the same axis as the epiproct (Fig. 1e, f). Length of ventral sclerite two-thirds of the epiproct; apical end with numerous, prominent spines. Dorsal sclerite in dorsal view with a strongly sclerotised, straight base. Lateral branches as long as the epiproct; ending in two slender, inwards-curved tips. Female and larva: unknown.

#### Diagnosis

*Protonemuraeclipsis* sp. nov. is distinguished from congeners by the distinct form of the inner, median and outer lobes of the paraprocts, as well as by the shape of the epiproct.

#### Etymology

Eclipsis is derived from the Greek root word ékleipsis, respectively the Latin root word eclipsis. In English, the term eclipse typically refers to the partial or complete obscuring of one celestial body by another or by the shadow of another, but is also associated with a broader range of meanings like darkness, obscurity or blackness. The specific name is a tribute to Pink Floyds song “Eclipse” and refers to the dark and prominent appearance of the paraprocts.

#### Affinities

The presence of a bifid terminal filament at the apex of the epiproct designates the new species as a member of the *Protonemuracorsicana* species group, *P.corsicana* subgroup (Vinçon and Murányi 2009). *Protonemuraeclipsis* sp. nov. is morphologically closely related to *Protonemuraalbanica*; however, it differs from *P.albanica* mainly by the shape of the paraprocts (Raušer 1963, fig. 2; Murányi 2007, figs. 79–82). While the outer lobe of the paraprocts bears two spikes on the outer projection in *P.albanica* (Fig. 2b), the outer lobe of the new species is missing such a structure (*Fig. 2a*). The shape of the median lobe is rounded in *P.albanica*, but is pointed and, thus, triangular-shaped in the new species. The inner expansion of the median lobe is slim and convex in *P.albanica* (Murányi 2007, figs. 79–82), while the new species bears a much more pronounced, ridge-like and concave structure with a variable number of prominent spines at the distal end. Furthermore, the distal endings of the lateral branches of the dorsal sclerite of the epiproct are straight in *P.albanica*, while they are pointed inwards in the new species (Fig. 2c, d). In lateral view, the lower ridge of the epiproct of *P.albanica* bears a hemispherical bulge with numerous spines (Raušer 1963, fig. 2F). In the new species, however, the spines insert directly on the inferior face of the epiproct, a hemispherical bulge is missing.

According to the previously mentioned characteristics of the four subgroups of the *P.corsicana* group (Vinçon and Murányi 2009), we propose to place the new species in the *P.corsicana* subgroup. The placement in the *P.corsicana* subgroup is not only justified by the considerable morphological similarities between *P.eclipsis* sp. nov. and *P.albanica*, but also by the shape of the ventral bulge of the epiproct. As opposed to a strongly pronounced ventral bulge in the three other subgroups, members of the *P.corsicana* subgroup are characterised by a slight ventral bulge of the epiproct, which also applies for the new species.

The new species differs from *Protonemuramiatchense* Ikonomov, 1983 by the shape of the paraprocts (Ikonomov 1983, figs. 18–22). While the inner lobe is slim and outwards-orientated in *P.miatchense*, the inner lobe of the new species is distally expanding before ending in an elongated, slender apex. Furthermore, the inner expansion of the median lobe is slim and single-pointed in *P.miatchense*, while the corresponding structure of the new species bears a variable number of prominent spines at the distal end. The terminal filament of the epiproct is much shorter in *Protonemuramiatchense* than in the new species.

##### Ecology and distribution of Protonemuraeclipsis and congeners

As indicated by Fig. 4, *P.albanica* was firstly recorded in Albania, District of Tepelenë, Uji i Ftohtë, as well as in Borshi south of Vlora (Raušer 1963). Topotypes were collected in similar localities by Murányi (2007) and the second author. Further specimens were found in the Kendrevicë Mountains near Progonat as well as in the Gjerë Mountains in the so-called Blue Eye Spring (Murányi 2007). The larvae are mainly associated with karst spring systems in altitudinal ranges between 50 and 200 metres above sea level (Raušer 1963, Murányi 2007). *P.miatchense*, however, is only known from further north, namely from three localities in Macedonia: the holotype was collected at the Mavrovska River close to the Bistra Mountains, additional specimens were collected in the mountains ranges of Jablanitsa and Karaorman. Larvae were collected in mountain rivers and small limestone rivers in habitats ranging between 900 and 1.400 metres above sea level (Ikonomov 1983). Both *Protonemuraalbanica* and *Protonemuramiatchense* are generally flying in spring; however, adult organisms of *P.albanica* have also been collected in October (Raušer 1963, Ikonomov 1983, Murányi 2007).

**Figure 3.**
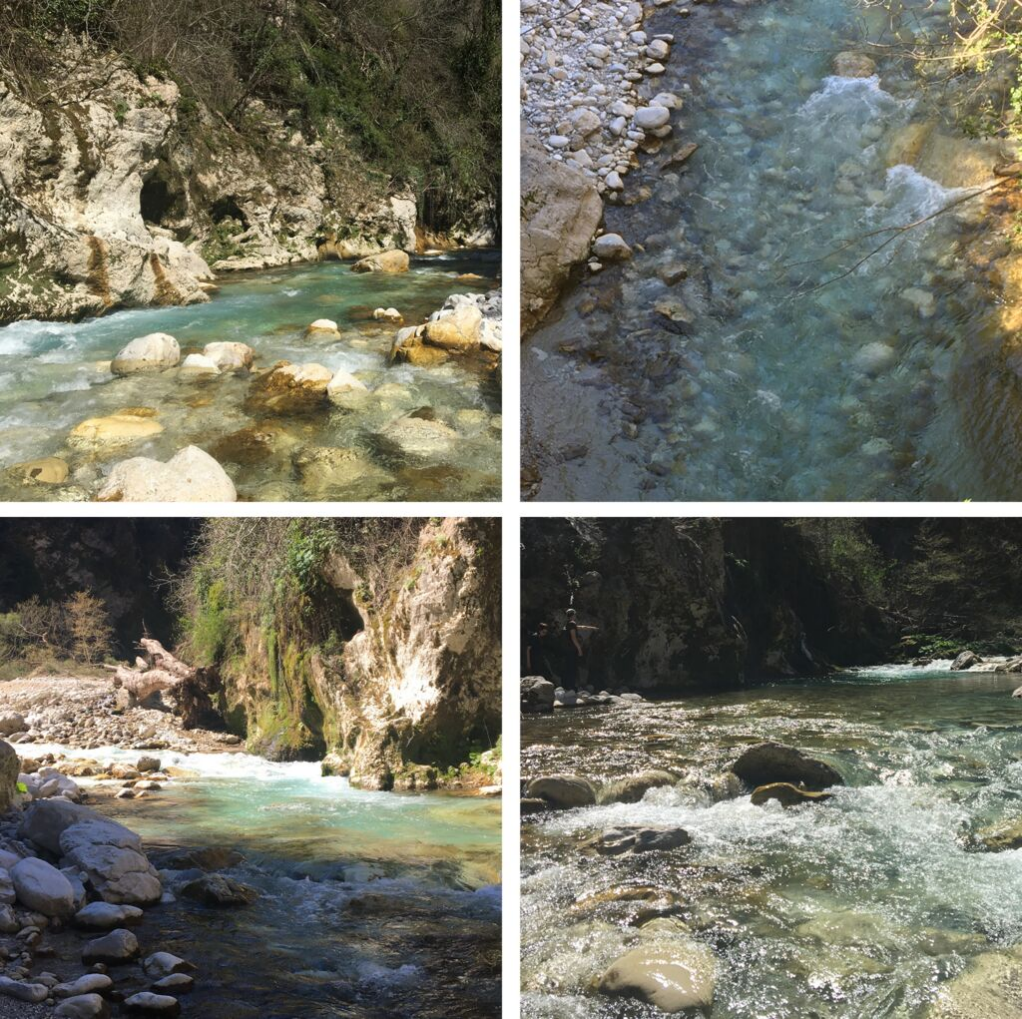
Locus typicus of *Protonemuraeclipsis* sp. nov. (Photos Mearie Wahl).

**Figure 4.**
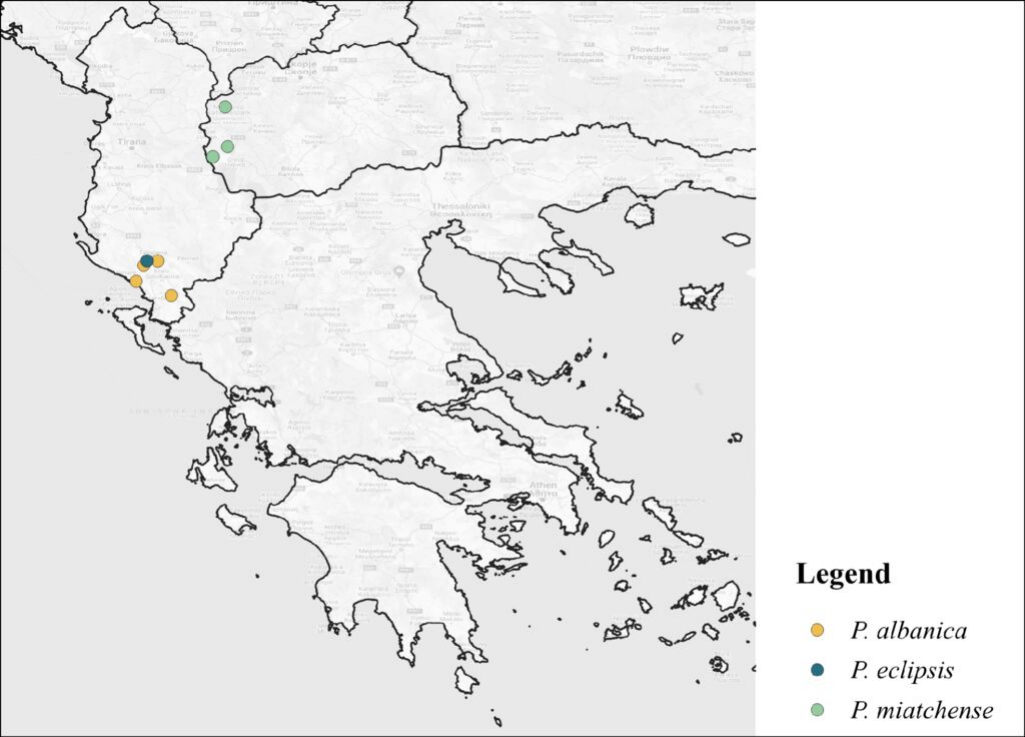
Distribution of *P.albanica, P.eclipsis* sp. nov. and *P.miatchense* (Raušer 1963, Ikonomov 1983, Murányi 2007).

As mentioned before, the holotype of *P.eclipsis* sp. nov. was found on the banks of the River Bënçë (Fig. 3), close to Tepelenë (Albania) at an altitude of 300 metres above sea level. As it was collected in March, it can also be designated as a spring species. The Bënçë presents itself as a turbulent mountain river with coarse substrate and a maximum temperature of 20.2°C (Graf, unpublished data). The macroinvertebrate fauna of the stream reflects a typical species community of a fast-flowing mountain streams, compromising species like the Plecoptera *Perlamarginata* and *Chloroperlatripunctata*, the Trichoptera *Rhyacophila nubila, Hydroptila brissaga, H.vectis, H. simulans, Psychomyia pusilla* and *Thremmaanomalum* and the Blephariceridae *Liponeurabilobata* (Graf, unpublished data).

## Supplementary Material

XML Treatment for Protonemura eclipsis

## Figures and Tables

**Figure 1a.**
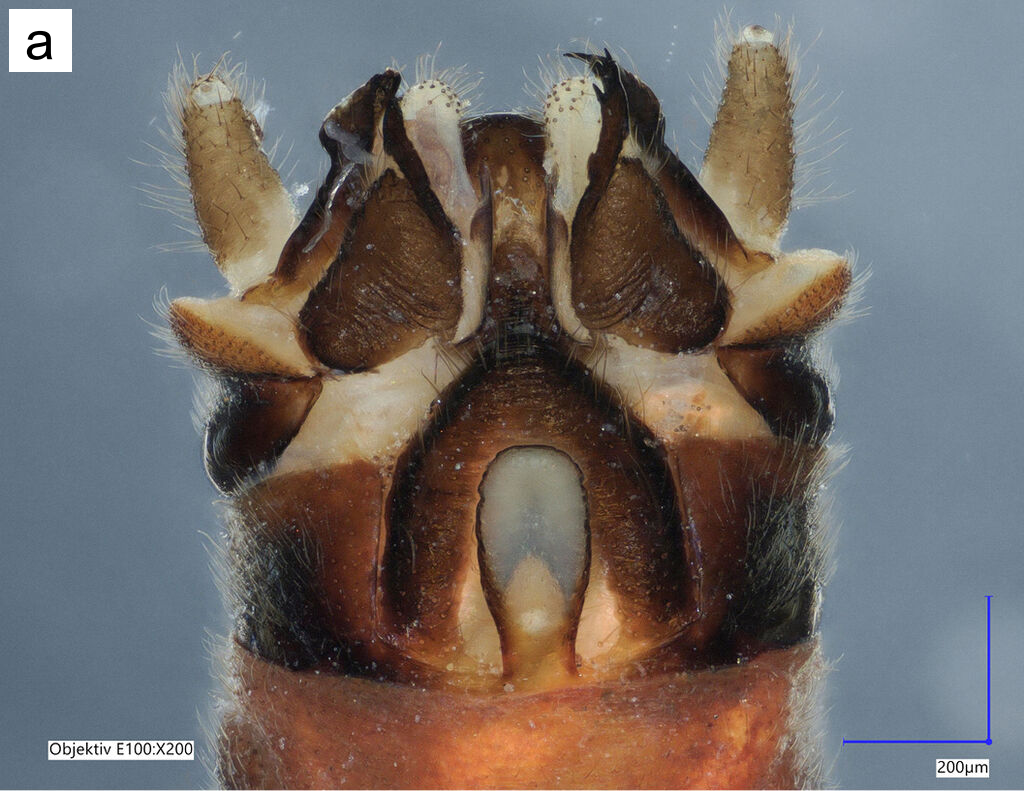
Ventral view;

**Figure 1b.**
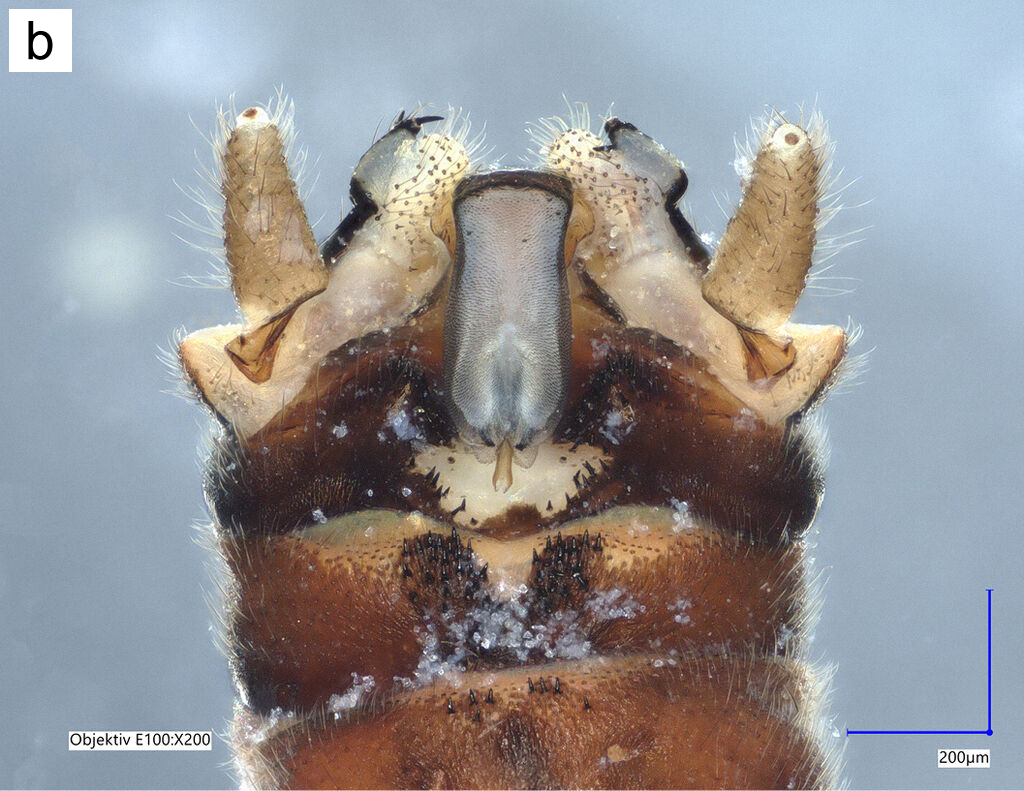
Dorsal view;

**Figure 1c.**
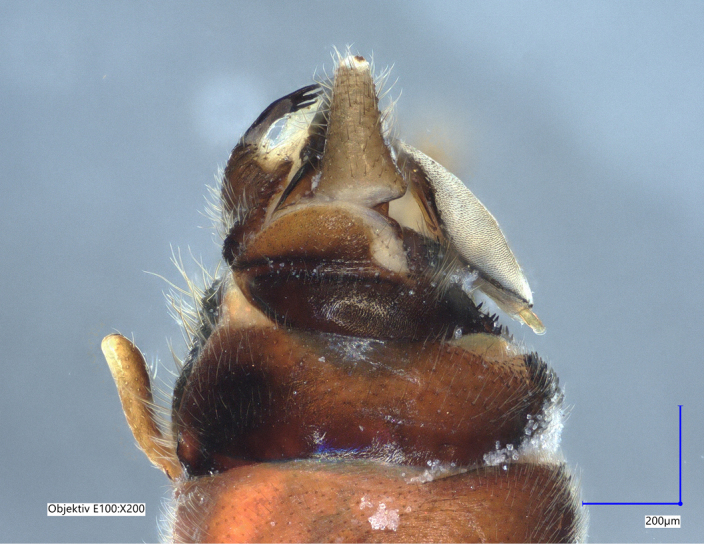
Left side, lateral view;

**Figure 1d.**
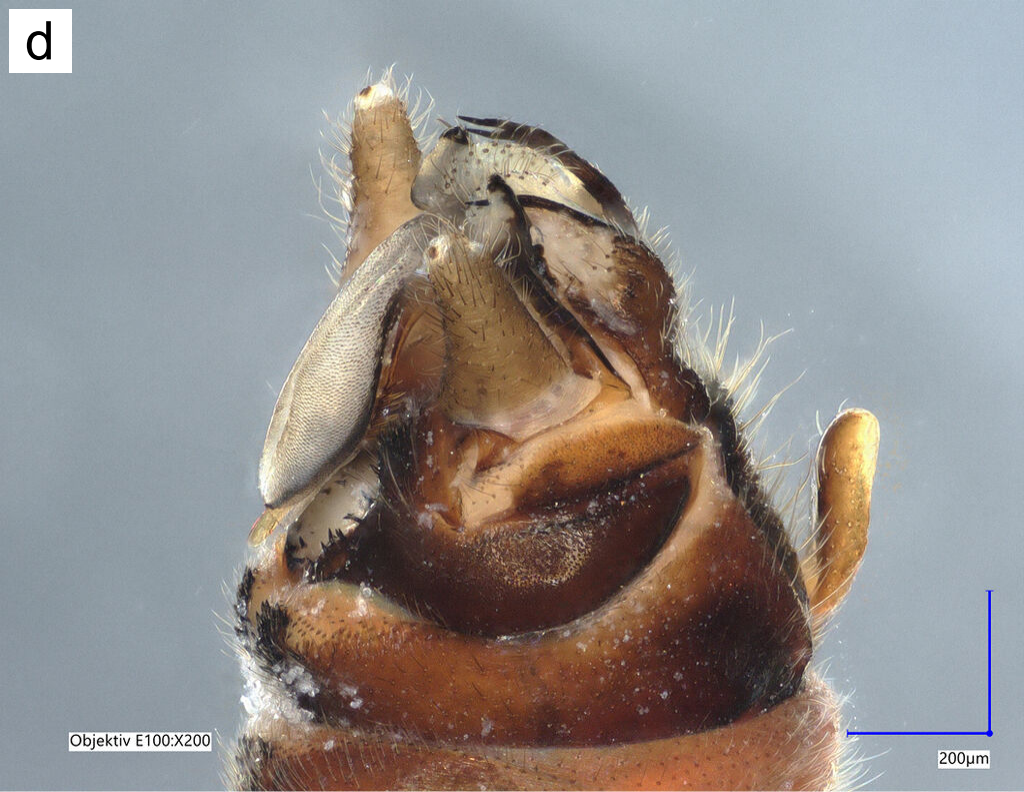
Right side, lateral view;

**Figure 1e.**
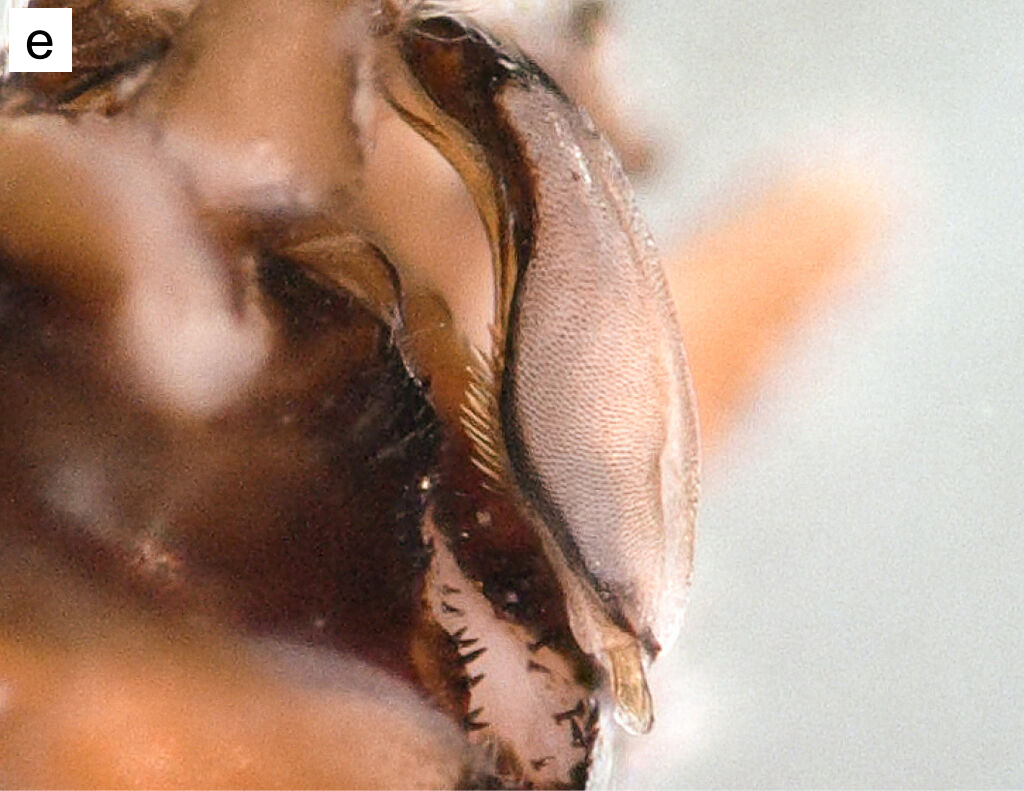
Epiproct left side, lateral view;

**Figure 1f.**
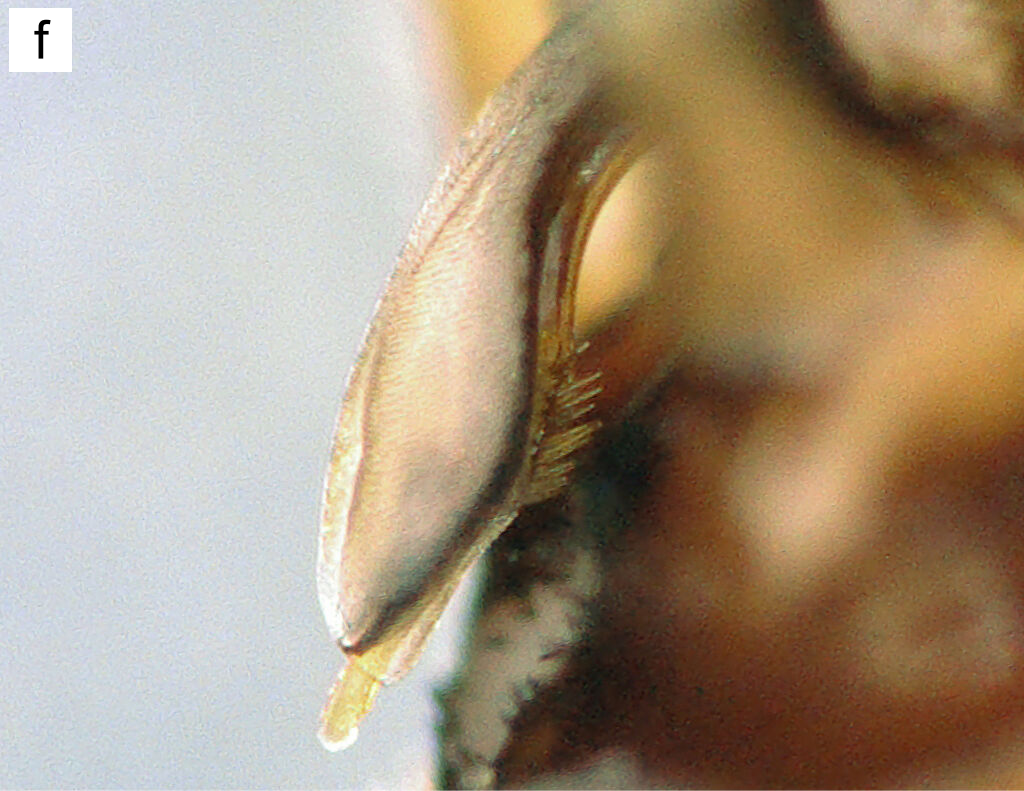
Epiproct right side, lateral view.

**Figure 2a.**
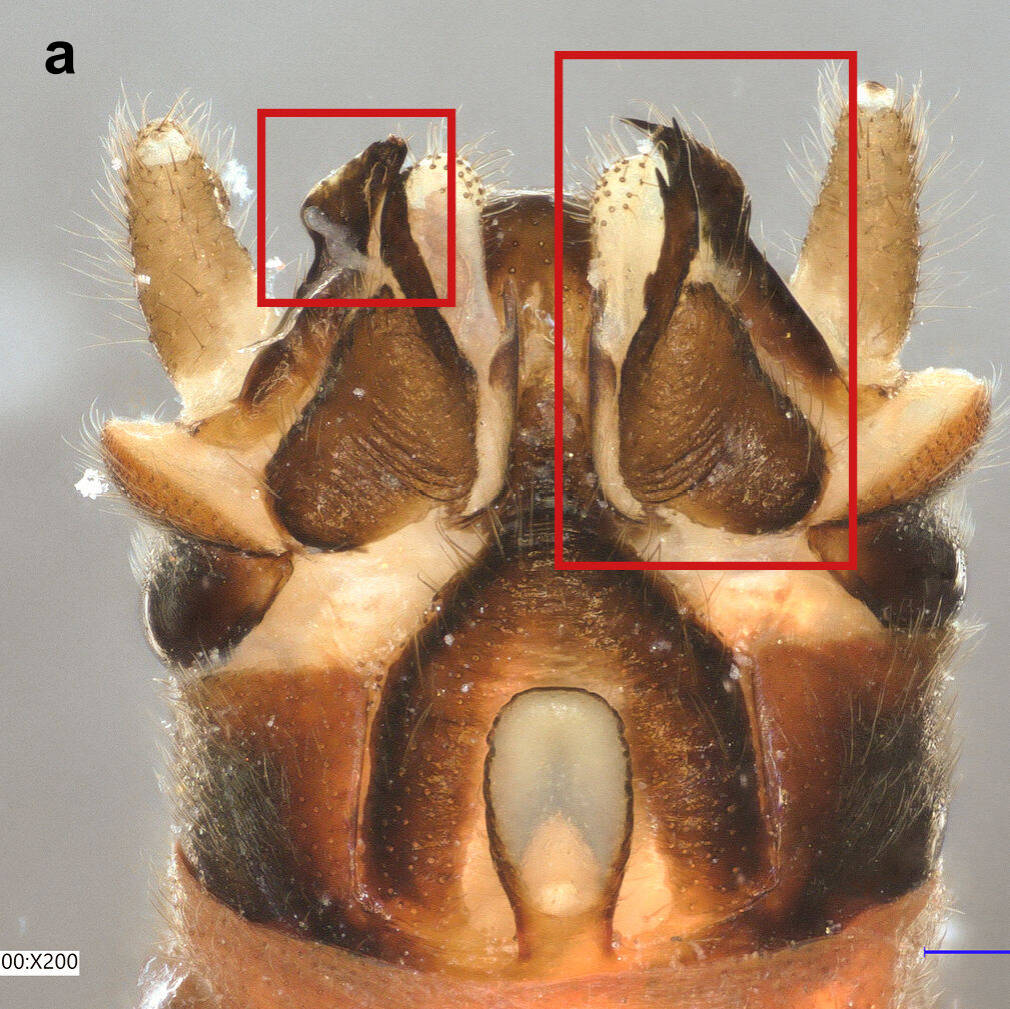
*Protonemuraeclipsis* sp. nov, male terminalia, ventral view;

**Figure 2b.**
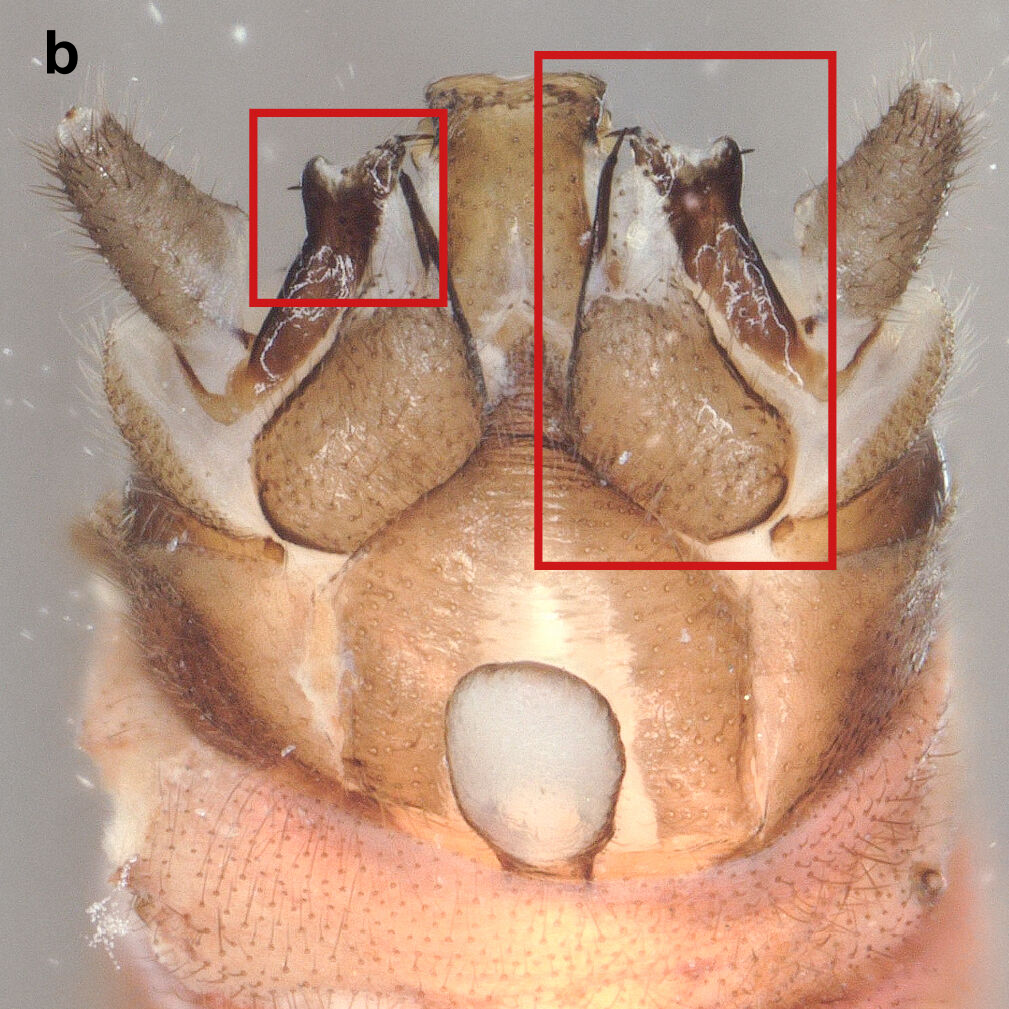
*Protonemuraalbanica*, male terminalia, ventral view;

**Figure 2c.**
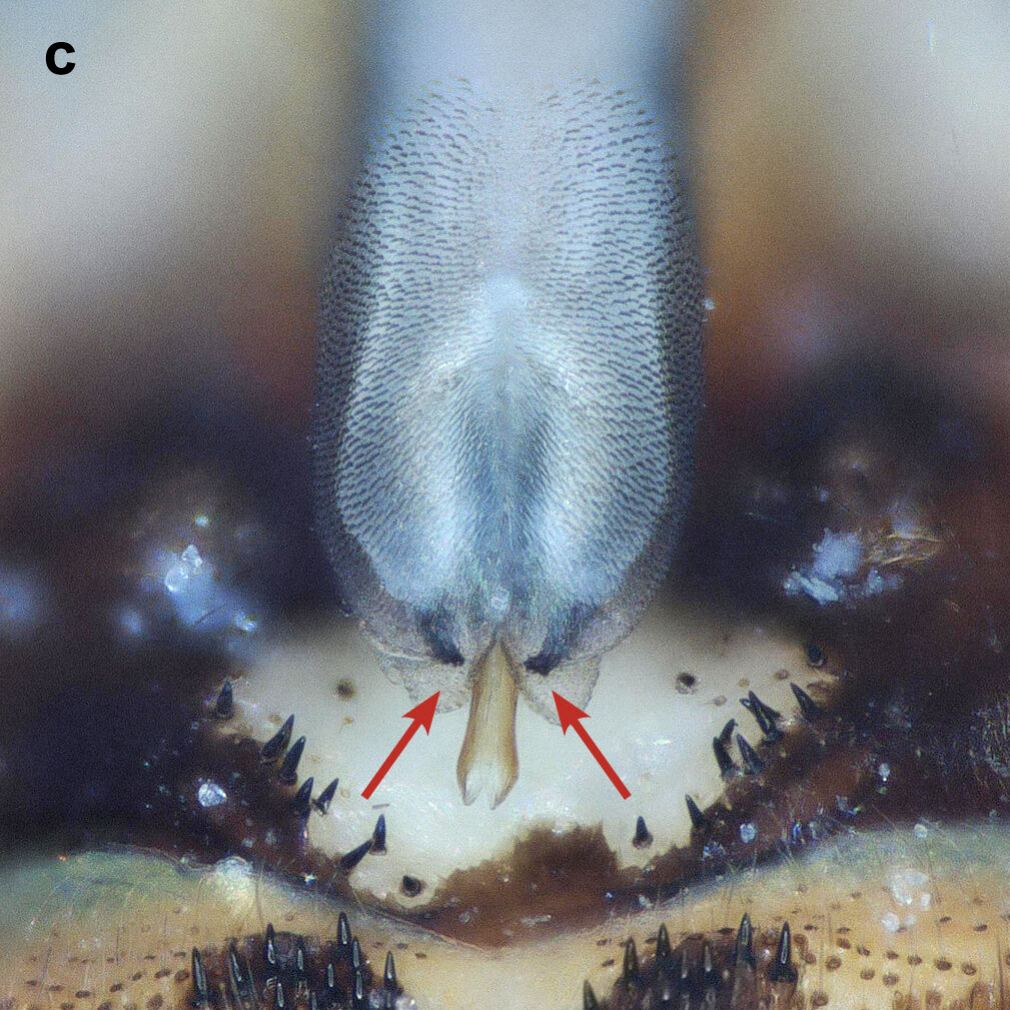
*Protonemuraeclipsis* sp. nov, epiproct;

**Figure 2d.**
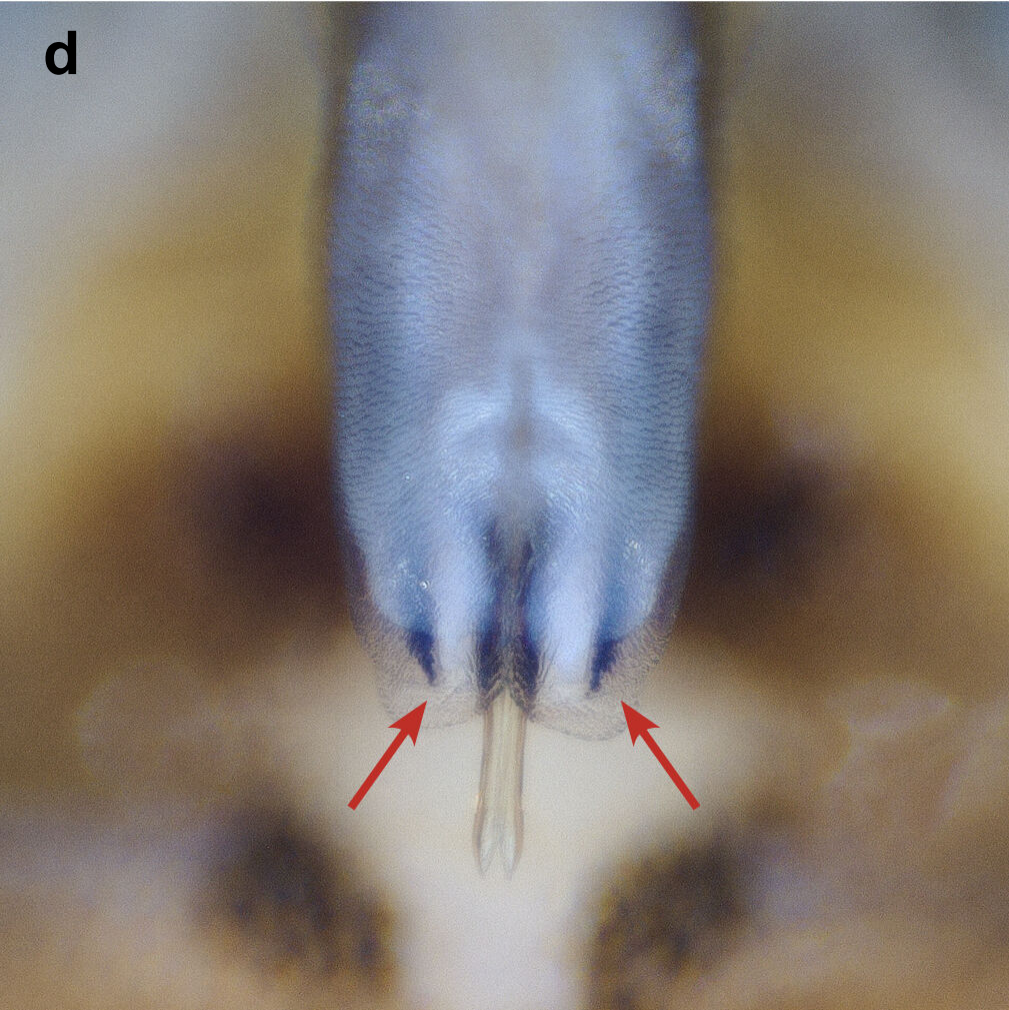
*Protonemuraalbanica*, epiproct.
